# Epidemiological Significance of the Fox (*Vulpes vulpes*) in the Spread of Vector-Transmitted Zoonoses in the Area of Northern Croatia

**DOI:** 10.3390/pathogens14090858

**Published:** 2025-08-29

**Authors:** Marina Pavlak, Jelena Prpić, Ioana A. Matei, Krešimir Trninić, Snježana Ćurković, Željko Mihaljević, Zrinka Štritof, Ksenija Vlahović, Žarko Udiljak, Lorena Jemeršić

**Affiliations:** 1 Department of Veterinary Economics and Epidemiology, Faculty of Veterinary Medicine, University of Zagreb, 10000 Zagreb, Croatia; 2 Laboratory for Diagnosis of Classical Swine Fever, Molecular Virology and Genetics (Z-III-1), Croatian Veterinary Institute, 10000 Zagreb, Croatia; jemersic@veinst.hr; 3 Department of Microbiology, Immunology and Epidemiology, Faculty of Veterinary Medicine, University of Agricultural Sciences and Veterinary Medicine Cluj-Napoca, 400372 Cluj-Napoca, Romania; ioana.matei@usamvcluj.ro; 4 The Paying Agency in Agriculture, Fisheries and Rural Development, 10000 Zagreb, Croatia; kresimir.trninic@apprrr.hr; 5 Department of Anatomy, Histology and Embryology, Faculty of Veterinary Medicine, University of Zagreb, 10000 Zagreb, Croatia; curkovic@vef.hr; 6 Pathology Laboratory (Z-IV-1), Croatian Veterinary Institute, 10000 Zagreb, Croatia; miha@veinst.hr; 7 Department of Microbiology and Infectious Disease, Faculty of Veterinary Medicine, University of Zagreb, 10000 Zagreb, Croatia; zstritof@vef.unizg.hr; 8 Department of Veterinary Biology, Faculty of Veterinary Medicine, University of Zagreb, 10000 Zagreb, Croatia; vlahovic@vef.hr; 9 Faculty of Dental Medicine and Health, Josip Juraj Strossmayer University of Osijek, 31000 Osijek, Croatia; ordinacija.zudiljak@gmail.com

**Keywords:** seroepidemiologic studies, zoonoses, risk factors, *Anaplasma phagocytophilum*, *Dirofilaria immitis*, *Borrelia burgdorferi*, molecular isolation, genetic identification, Croatia

## Abstract

Wild animals often serve as reservoirs for vector-borne zoonoses, which are on the rise worldwide but have not yet been sufficiently researched. Vector-borne zoonoses, such as those caused by *Anaplasma phagocytophilum*, *Borrelia burgdorferi* sensu lato, and *Dirofilaria immitis*, are a growing public health concern due to their increasing incidence and broad host range. The aim of this study was to determine the prevalence and risk factors for vector-borne bacterial (borreliosis, anaplasmosis, ehrlichiosis) and parasitic (dirofilariasis) pathogens and to detect some of these pathogens in the red fox (*Vulpes vulpes*) population in Croatia. A total of 179 blood samples from foxes from nine districts were analysed. The SNAP ^®^ 4Dx ^®^ Plus rapid test was used to detect circulating *D. immitis* antigen and antibodies against *B. burgdorferi*, *A. phagocytophilum*/*Anaplasma platys*, and *Ehrlichia canis*/*Ehrlichia ewingii*. Circulating *D. immitis* antigen was detected in 6.70% of the samples (95% CI: 3.20–10.19%), while antibodies against *A. phagocytophilum*/*A. platys* were found in 10.06% (95% CI: 5.8–14.25%). Only one sample was positive for *B. burgdorferi*, while no antibodies were detected for *E. canis*/*E. ewingii*. Spatial analysis revealed statistically significant differences in prevalence by geographical region (district) and age, while no significant correlations were found. In the standard PCR analysis, DNA of *D. immitis* was not detected in any of the eight positive and eight negative SNAP ^®^ 4Dx ^®^ Plus samples. *D. repens*, *A. reconditum*, or co-infections were also not detected by PCR. Of the nine samples that tested positive for *A. phagocytophilum*/*A. platys* antibodies, four were confirmed to be positive for *A. phagocytophilum* by nested and semi-nested PCR targeting the 16S rRNA and GroEL genes. Phylogenetic analysis revealed similarities with various European strains, including zoonotic strains. This study is the first molecular detection of *A. phagocytophilum* from blood samples of red foxes in Croatia. The results show that red foxes are not free from infections such as anaplasmosis and dirofilariasis, emphasising their possible role in the maintenance and transmission of these pathogens in certain regions of Croatia. These results underline the need for further research to better understand the epidemiological importance of red foxes in the spread of vector-borne diseases.

## 1. Introduction

Vector-borne zoonoses are infectious diseases that are naturally transmitted from animals to humans by vectors—usually ticks or mosquitoes. In recent years, the increased incidence of vector-borne zoonoses in humans and animals has become a major public health concern [1,2,3,4]. These zoonoses are categorised as emerging or re-emerging infectious diseases [3,5]. Ecological changes, especially due to climate change, influence the dynamics, population density, and geographical distribution of different vector species, which in turn affects the occurrence and spread of vector-borne zoonoses in humans and animals [6,7,8,9,10,11]. Among the pathogens of emerging tick-borne diseases, the intracellular bacteria *A. phagocytophilum* and *E. canis* and the spirochete *B. burgdorferi* are of particular importance due to their widespread distribution and zoonotic potential [3,5,7,9].

*A. phagocytophilum* is a Gram-negative, small, mostly pleomorphic, coccoid to ellipsoid bacterium that is transmitted by ticks of the *Ixodes* complex (in Europe, the main vector is *Ixodes ricinus*) [12]. It parasitises in neutrophil granulocytes and causes a disease known as granulocytic anaplasmosis [9,13]. Symptoms of anaplasmosis include fever, chills, headache, muscle (myalgia) and joint (arthralgia) pain, nausea, vomiting, weakness, and malaise [3]. The various possible reservoir species include rodents, deer (*Cervus elaphus*), wild boar (*Sus scrofa*), and red foxes (*Vulpes vulpes*), while horses, dogs, and cats are recognised as domestic animals with potentially zoonotic genetic variants [12,14,15,16,17]. Phylogenetic analyses have shown that *A. phagocytophilum* has considerable genetic diversity, including variations in genes such as groEL, ankA, and msp4 [12]. Epidemiological studies in Europe have shown an increased occupational risk for forest workers, hunters, veterinarians, and farmers who are frequently exposed to tick bites in endemic areas [12,14].

Throughout Europe and the northern hemisphere, the most common tick-borne pathogens are spirochetes of the *B. burgdorferi* sensu lato (s.l.) complex, which cause Lyme borreliosis. This disease can affect multiple organs, including the heart, nervous system, joints, and skin [18,19]. Reservoir hosts of *B. burgdorferi* in Europe include the European hedgehog (*Erinaceus europaeus*), voles, and mice (*Clethrionomys glareolus*, *Apodemus sylvaticus*, *Apodemus flavicollis*) as well as the red fox (*Vulpes vulpes*) and the bridled deer (*Rangifer tarandus*). Domestic animals such as cattle, horses, and dogs can also be included in the cycle and serve as hosts for infected ticks. Dogs and horses are also susceptible to Lyme disease themselves and symptoms such as lameness, fever, inappetence, lethargy, and focal lymphadenopathy show in dogs and neurological abnormalities, uveitis, and cutaneous lymphoma show in horses. Interspecies transmission is mainly associated with the life cycle of ticks of the genus *Ixodes* [20]. In many parts of Europe, where the pathogen is endemic in animals, these can serve as indicators of infection [21] and also as sources of human infection, mainly manifesting as neuroborreliosis [22,23]. Some studies suggest a possible link between infection with *B. burgdorferi* and the aetiology of multiple sclerosis [24].

*D. immitis* is a parasitic roundworm that infects various animals—mainly carnivorous canids and felids—as well as humans [25]. It is the causative agent of canine heartworm disease (cardiopulmonary dirofilariasis), which leads to various symptoms that are mainly due to circulatory disorders. A common symptom in dogs is a persistent, chronic, nonproductive cough, which worsens with exertion, followed by moderate or severe dyspnoea and/or tachypnoea on exertion, including a rise in body temperature, abnormal heart and lung sounds, syncope, hepatomegaly, epistaxis, ascites, loss of appetite, and weight loss [26,27,28]. Mosquitoes from the Culicidae family serve as intermediate hosts. A major public health concern is that infected dogs often remain asymptomatic, while they represent a reservoir for transmission of the pathogen to humans.

The red fox (*Vulpes vulpes*), a wild canid species, is considered a potential reservoir for several vector-borne diseases [29,30,31,32,33,34,35,36,37,38,39]. It is highly adaptable to the human-altered environment and is widely distributed in rural and suburban areas [31]. The role of foxes in the transmission of emerging and re-emerging infectious diseases, including vector-borne diseases, is increasingly recognised [40,41,42,43,44]. However, their exact role as reservoirs for these pathogens is not yet fully understood. Although molecular and immunological techniques have improved the detection of infections in both domestic and wild animals, including foxes, further research is needed to fully elucidate their epidemiological impact [45].

In Croatia, seroprevalence studies on vector-borne diseases have mainly focused on humans—especially those with Lyme disease [46,47,48,49,50,51,52,53]—and domestic animals, particularly dogs [54,55,56,57,58] and cattle [59], as well as ticks [60,61].

The aim of the present study is to investigate and evaluate the presence, prevalence, and geographical distribution of vector-borne zoonotic pathogens such as *D. immitis*, *A. phagocytophilum*, *B. burgdorferi*, and *E. ewingii* in red foxes in order to assess their role in the epidemiology of these diseases in Croatia. In addition, this study aims to determine the potential role of red foxes as sentinel or reservoir hosts, especially in regions where human and domestic animal exposure to vectors is high, and to analyse the genetic diversity of the detected pathogens to better understand their zoonotic potential and evolutionary relationships.

## 2. Materials and Methods

### 2.1. The Study Population

This study was conducted with a population of red foxes (Vulpes vulpes) in northern Croatia, which were delivered to the Croatian Veterinary Institute in Zagreb to control the immunity of oral rabies vaccination, based on the Regulation on measures for the protection of animal health against infectious diseases and their financing in 2020 and 2021. This study was approved by the Ethics Committee of the Faculty of Veterinary Medicine of University of Zagreb (Ur. Broj 251-61-44/168-18/21, Klasa 640-01/18-17/01, 12 July 2018). Before sampling, all foxes were tested for rabies and only foxes that tested negative were sampled. Although it could be argued that the samples taken in this way were technically opportunity samples, it is important to emphasise that the samples were taken randomly in the field and in different regions, providing a degree of geographical diversity and representativeness, which reduces the potential for significant bias and allows for partial generalisation of the results to the fox population in the region where the samples were taken.

The red fox (*Vulpes vulpes*) was sampled in nine districts in northern Croatia, covering a total of 127 hunting areas. These regions cover the approximate geographical coordinates of 45.1° to 46.3° north latitude and 13.8° to 16.5° east longitude, including places such as Varaždin, Karlovac, Rijeka, and Zagreb. The climate in these areas is categorised as temperate continental, with maritime and mountainous influences depending on altitude and proximity to the Adriatic coast. During the sampling period (2020–2021), average annual temperatures ranged from 7.4 °C to 17.5 °C, with maximum temperatures reaching up to 30 °C in summer and minimum temperatures falling below −5 °C in winter. Relative humidity generally ranged between 60% and 74%, and annual rainfall varied between 780 mm and 880 mm throughout. These climatic conditions are consistent with the ecological requirements of red fox populations and may influence the prevalence and transmission dynamics of vector-borne pathogens.

The foxes were of both sexes and between a few months and seven years old. Blood samples were taken at necropsy from the thoracic cavity, mainly from the heart, and collected in EDTA-containing tubes. Immediately after collection, a SNAP ^®^ 4Dx ^®^ Plus test (SNAP ^®^ Test, IDEXX, Westbrook, ME, USA) was performed, while the remaining amount of collected blood was stored at −20 °C for molecular diagnostic testing using standard PCR and nested PCR methods. Standard PCR reactions were used to amplify the target DNA of D. immitis [62] and nested and semi-nested PCR reactions were used to amplify regions within the 16S rRNA and GroEL segments of the *A. phagocytophilum* genome [63,64]. Nested and semi-nested PCR reactions were used to amplify a specific DNA region isolated from fox ear tissue encoding the OspA protein of the bacterium B. burgdorferi [65].

### 2.2. SNAP ^®^ 4Dx ^®^ Plus Test

The SNAP ^®^ 4Dx ^®^ Plus screening test for the detection of circulating D. immitis antigen, antibodies of *B. burgdorferi*, *A. phagocytophilum*/*A. platys*, and antibodies of *E. canis*/*E. ewingii* was used according to the manufacturer’s instructions (IDEXX, SNAP ^®^ Test Quick Reference Guide). The result is positive if a colour appears at the points marked for a particular pathogen and negative if a colour appears only at the positive control point.

### 2.3. Molecular Analysis

DNA was extracted from 200 µL of EDTA- frozen blood using the commercial kit ReliaPrep™ Blood gDNA Miniprep System (Promega, Madison, WI, USA) according to the manufacturers’ instructions and stored at −20 °C until analysis.

### 2.4. PCR for the Amplification of the Target Part of the DNA of Dirofilaria immitis Species

All samples were analysed for detecting *Dirofilaria* DNA using the target gene 5.8S-ITS2-28S rDNA. After the initial screening, the primers DIDR-F1 F1 (F1 5′-AGTGCGAATTGCAGA CGCATTGAG-3′) and DIDR-R1 (5′-AGCGGGTAATCACGACTGAGTTGA-3′) (Sigma Aldrich, Saint Louis, MO, USA) were used for the PCR amplification reaction of *D. immitis* from positive samples [62]. The PCR reaction was performed in a final volume of 50 µL using GoTaq ^®^ Green Master Mix (Promega, Madison, WI, USA). Cycling reactions were performed using the GenAmp System 9700 (Applied Biosystems, Carlsbad, CA, USA) under the following conditions: initial activation of the polymerase at 95 °C for two minutes, followed by 32 cycles of chain separation at 95 °C for 30 s, primer pairing at 60 °C for 30 s, and primer extension at 72 °C for 30 s. The final elongation of the amplified DNA sequences was carried out at 72 °C for seven minutes. Each PCR reaction set contained positive and negative controls. All amplification products were visualised by electrophoresis in a 2% agarose gel (1 × TAE, pH 8.0) stained with a DNA gel dye (Diamond ^TM^ Nucleic Acid Day, Promega, Madison, WI, USA). A marker for the determination of double-stranded DNA molecules in the size range from 100 to 1500 pb (BenchTop 100 bp DNA Ladder, Promega) was also used to determine the product size.

### 2.5. Nested and Semi-Nested PCR Reactions for the Amplification of Regions Within the 16S rRNA and GroEL Segments of the A. phagocytophilum Genome

For the amplification of regions within the 16S rRNA segment of the *A. phagocytophilum* genome, a nested PCR [63] with two primer pairs was used: the outer primer pair ge3a (5′-CACATGCAAGTCGAACGGATTATTC-3′) and ge10r (5′-TTCCGTTAAGAAGGATCTAATCTCC-3′) (Sigma Aldrich, Saint Louis, MO, USA) and the inner primer pair ge9f (5′-AACGGATTATTCTTTATAGCTTGCT-3′) and ge2 (5′-GGCAGTATTAAAAGCAGCTCCAGG-3′) (Sigma Aldrich, Saint Louis, MO, USA). Direct PCR was performed using the primer pair ge3a/ge10r [63] to amplify a 932 bp fragment using a commercial ALLin ^TM^ Red Taq Mastermix, 2 x (highQu) kit. The PCR reaction was performed using the Gene Amp PCR System 9700 (Applied Biosystems, Carlsbad, CA, USA). Since the amplified fragments of 932 bp were not visible after electrophoretic analysis in a 1.5% agarose gel, they were used as a template for nested PCR with the primer pair ge9f/ge2 [63]. The expected nested PCR product was a fragment of 546 bp which was amplified with the commercial ALLin Red Taq Mastermix, 2 x (highQu) kit.

Semi-nested PCR reactions [64] were used to amplify regions within the GroEL segment of the *A. phagocytophilum* genome containing the outer primer pair EphplgroEL(569)F (5′-ATGGTATGCAGTTTGATCGC-3′) and EphplgroEL(1193)R (5′-TCTACTCTGTCTTTGCGTTC-3′) and the inner primer pair EphplgroEL(569)F (5′-ATGGTATGCAGTTTGATCGC-3′) and EphgroEL(1142)R (5′-TTGAGTACAGCAACACCACCGGAA-3′). The first PCR reaction was performed with the primer pair EphplgroEL(569)F/EphplgroEL(1193)R [64] to amplify a 750 bp fragment using a commercially available kit, ALLin ^TM^ Red Taq Mastermix, 2 x (highQu). The PCR cycles were performed using a Gene Amp PCR System 9700 (Applied Biosystems, Carlsbad, CA, USA). The amplified segments were used as templates for a semi-nested PCR with the primer pair EphplgroEL(569)F/EphgroEL(1142)R [64], where a fragment of 573 bp in size was amplified with the commercial kit ALLin Red Taq Mastermix, 2 x (highQu). DNA isolates previously confirmed positive for *A. phagocytophilum* were used as positive controls. The negative controls were aliquots of ultrapure water.

All confirmed nested positive amplicons in the agarose gel revelation were purified using the Wizard ^®^ SV Gel and PCR Clean-UP System (Promega, Madison, WI, USA). The purified PCR products were sent to Macrogen Inc. (Amsterdam, The Netherlands) for sequencing. To determine the nucleotide sequence, the chromatograms obtained were analysed using the Sequencher 5.4.6 programme (http://www.genecodes.com, accessed on 19 August 2025) Genes Codes Corporation). The following programmes and software packages were used: for computer analysis of the sequences obtained, ClustalX, version 1.83 [66]; for multiple alignment of sequences to find the location of changes/mutations in the analysed nucleotide sequences and to prepare the data for phylogenetic analysis, BioEdit, version 7.0.5.3 [67]; for editing aligned sequences, MEGA, version [68]; for phylogenetic and molecular evolutionary analysis using different methods and procedures, BLAST *+* 2.17.0 [69]; and for searching databases, http://www.ncbi.nlm.nih.gov, accessed on 19 August 2025.

### 2.6. Nested and Semi-Nested PCR Reaction for the Amplification of B. burgdorferi

Ear punch biopsies from one SNAP-positive fox and from twenty randomly selected foxes that tested negative for Borrelia using the SNAP test were analysed using a nested PCR reaction performed in two parts, using two different primer pairs in two separate reactions. In the external PCR reaction, a specific DNA segment coding for the OspA protein of the bacterium B. burgdorferi was amplified with the primers N1 5′GAGCTTAAAGGAACTTCTGATAA-3′ and C1 5′-GTATTGTTGTACTGTAATTGT-3′ [65]. The PCR product obtained served as a template for an internal PCR reaction with the primers N2 5′-ATGGATCTGGAGTACTTGAA-3′ and C2 5′-CTTAAAGTAACAGTTCCTTCT-3′ [65]. The PCR reaction was carried out in a final volume of 25 µL Emerald PCR mix.

### 2.7. Statistical Analysis

Percentages with 95% confidence intervals (CI 95%) were used for the catechetical variables. Statistically significant differences in seroprevalence between groups were determined using the chi-square statistic when more than two groups were included and Fisher’s exact test for two-way comparisons only. To assess the strength of the association between infection and the risk factors, including geographical location (districts), age and sex, the odds ratio (OR), and the corresponding 95% confidence interval were calculated using logistic regression analysis [70]. The age cut-off for distinguishing between young and adult foxes was 1 year [71,72]. Significance between groups was assumed at the 5% level (*p* < 0.05). All statistical and epidemiological analyses were performed using STATISTICA 12 and WinEpiscope software. 2.

## 3. Results

### 3.1. Results of the Examination with the SNAP ^®^ 4Dx ^®^ Plus Test

**Figure 1:**
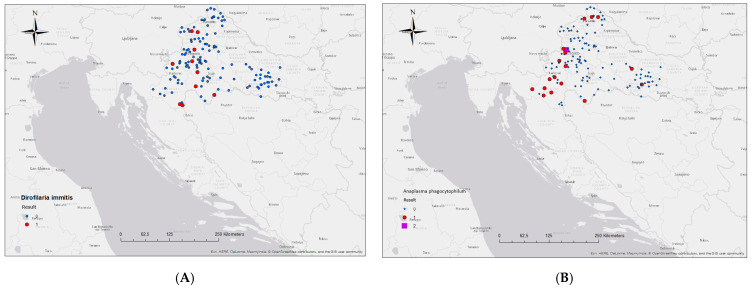
Spatial distribution of the prevalence of *D. immitis* (**A**) and the seroprevalence of *A. phagocytophilum* (**B**) in northern Croatia. The blue dots show all tested foxes with a negative screening test, while the red dots indicate positive foxes.

Of the 179 foxes tested, circulating *D. immitis* antigen was found in 6.70% (CI 95%, 3.47–11.71%) and antibodies against *A. phagocytophilum*/*A. platys* in 10.06% (CI 95%, 5.96–15.89%) of the foxes. Although the foxes were exposed to 1.63 times more *Anaplasma* than *Dirofilariae*, the difference in prevalence was not statistically significant (*p* = 0.2526; χ^2^ = 1.309). Only one sample was positive for *B. burgdorferi*, while no antibodies were detected for *E. canis*/*E. ewingii.* The number of tested and positive foxes by area is shown in Figure 1.

Antibodies for *A. phagocytophilum*/*A. platys* were detected in foxes from six districts analysed, ranging from 4.55% (CI 95%, 0.16–25.33%) in the Požega-Slavonia County to 25.00% (CI 95%, 0.63–100.00%) in the Bjelovar-Bilogora County. These differences were not statistically significant. However, the lowest prevalence of *D. immitis* was found in Zagreb County (1.89%; CI 95%, 0.05–10.51%) compared to the other counties and was statistically significant (*p* < 0.05). The prevalence of *D. immitis* in foxes in other counties ranged from 4.55% (CI 95%, 0.12–25.33%) (Požega-Slavonia County) to 25.00% (CI 95%, 0.63–100.00%) (City of Zagreb). In comparison to the results of the negative *Anaplasma* test in foxes from the City of Zagreb and Krapina-Zagorje County, the *D. immitis* antigen was found in this area. In the northern areas, in the counties of Varaždin, Međimurje, and Bjelovar-Bilogora, *D. immitis* was not found (Table 1).

Sex was determined in 175 individuals, of which 108 (59.78%) were male and 67 (37.43%) female. No statistically significant differences between the sexes were found in the positive results for *A. phagocytophilum/A. platys* (14.93% (CI 95%, 6.39–23.46%) for females compared to 6.48% (CI 95%, 1.13–11.12%) for male foxes; *p* = 0.0673) or *D. immitis* (7.46% (CI 95%, 1.19–13.79%) for female vs. 6.48% (CI 95%, 1.13–11.12%) for male foxes; *p* = 0.08036).

Age was determined in 133 individuals, of which 71 (53.38%) were juveniles. The differences in seropositive results between age groups were not significant for *Anaplasma* (9.86% (CI 95%, 3.96–20.31%) for juvenile foxes vs. 12.90% (CI 95%, 5.57–25,42%) for adult foxes; *p* = 0.5818), but for *D. immitis* a statistically significantly higher prevalence was found in juvenile foxes (11.27% (CI 95%, 4.86–22.20%) vs. 1.61% (CI 95%, 0.04–8.99%) in adult foxes; *p* = 0.0275).

Regression analysis was used to analyse geographical area, sex, and age as risk factors. The results of the logistic regression analysis are summarised in Table 2 and Table 3. The results of the regression analysis show a possible correlation between the prevalence of *A. phagocytophilum* and *D. immitis* and potential risk factors, but no statistically significant correlation was found.

### 3.2. Results of the Molecular Protocols

All serologically positive samples that were suitable for molecular diagnostics were subjected to molecular analysis. However, as the blood was taken from dead animals, the quantity of samples obtained was insufficient in some cases. Therefore, out of 12 SNAP-positive samples for *D. immitis* and the 18 seropositive samples for *A. hagocytophilum*, eight and nine samples, respectively, were subjected to molecular analysis.

The standard PCR method for detecting the target gene 5.8S-ITS2-28S rDNA filaria of *D. immitis* was performed on the eight samples from foxes that tested positive for *D. immitis* using the SNAP ^®^ 4Dx ^®^ Plus test. Analysis of the PCR products amplified from the target DNA showed that none of the eight samples were actually positive. Similarly, neither the presence of *D. repens* or *A. reconditum* nor the presence of a possible co-infection could be detected using the standard PCR method.

**Figure 2:**
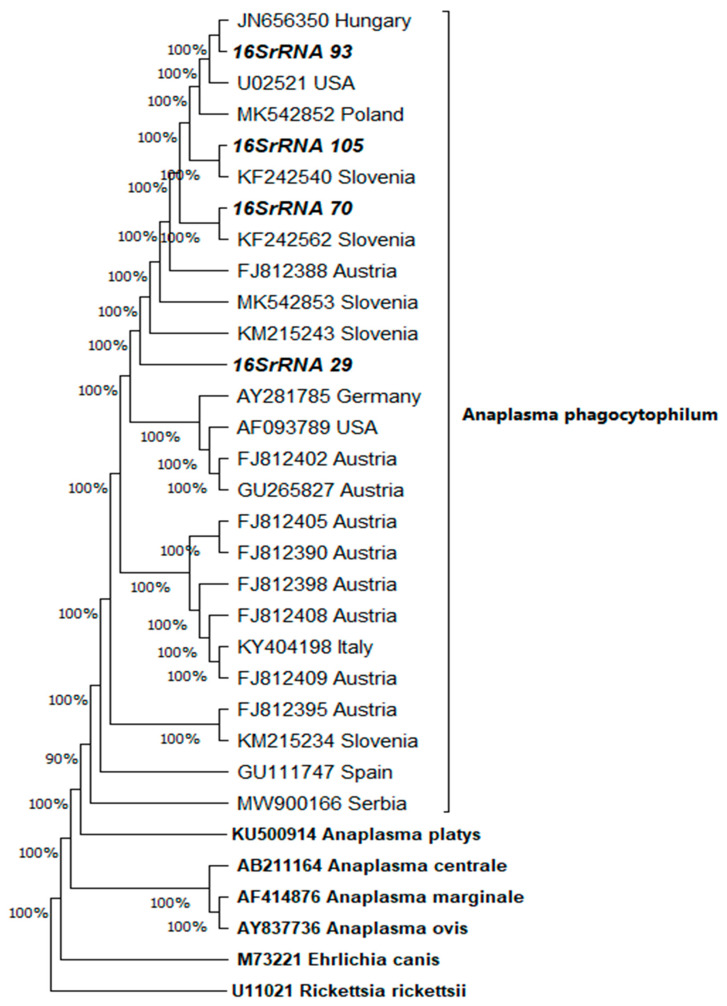
Phylogenetic tree representation of amplified 16S rRNA fragments (fragments 29, 70, 93, and 105 were amplified) constructed by neighbour-joining using the Kimura-2-parameter model. The numbers at the branch nodes represent bootstrap values in 1000 replicates. Related sequences were found using the BLAST algorithm.

**Figure 3:**
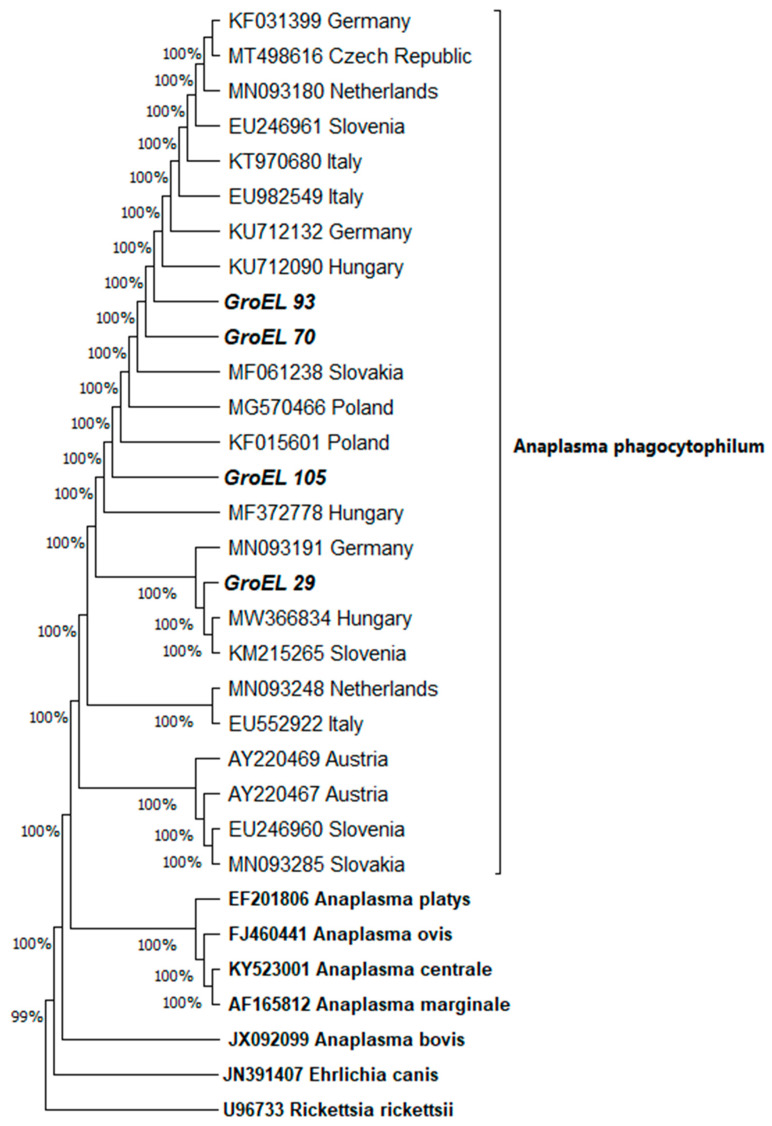
Phylogenetic tree representation of the amplified GroEL segments (segments for samples 29, 70, 93, and 105 were amplified) constructed by neighbour-joining using the Kimura-2-parameter model. The numbers in the branch nodes represent the values obtained by the bootstrap statistical method in 1000 repetitions. Related sequences were found using the BLAST algorithm.

Of the nine samples from foxes that tested positive *for A. phagocytophilum* using the SNAP ^®^ 4Dx ^®^ Plus test, four samples tested positive by amplification of segments of the 16S rRNA and GroEL genome of *A. phagocytophilum*. Phylogenetic trees for amplified segments of 16S rRNA (Figure 2) and GroEL (Figure 3) were constructed by the neighbour-joining method using the Kimura-2 parameter model for samples 29, 70, 93, and 105. Related sequences found using the BLAST algorithm were included in the analysis. The numbers in the branch nodes represented the values obtained by the statistical bootstrap method in 1000 replicates. Isolate 16S rRNA-29 showed the highest similarity with isolate MK542852 (human isolate from Poland), from which it differed in two nucleotides in the 491-nucleotide region; isolate 16S rRNA-70 with isolate KF242562 (isolate from wild boar from Slovenia), from which it differed by one nucleotide in the 491 nucleotide region; isolate 16S rRNA-93 with isolate JN656350 (dog isolate from Hungary), with which it was identical; and isolate 16S rRNA-105 with isolate KF242540 (dog isolate from Slovenia), from which it differed by one nucleotide in the 491 nucleotide region. Isolate GroEL-29 showed the greatest similarity with isolate MW366834 (isolate of Ixodes ricinus from Hungary), from which it differed by two nucleotides in the 445 nucleotide region; isolates GroEL-70 and GroEL-93 with isolates KU712090 (isolate from dog from Hungary) and MF061238 (isolate from domestic pig from Slovakia), with which they were identical; and isolate GroEL-105 with isolate KF015601 (isolate from human from Poland), from which it differed by 13 nucleotides in 445 nucleotide regions.

The molecular analyses carried out showed no evidence of *B. burgdorferi.*

## 4. Discussion

The results of the seroprevalence study of vector-borne diseases in foxes in northern Croatia showed the presence of antibodies against *A. phagocytophilum*/*A.platys* (10.06%; CI 95%, 5.96–15.89%) and circulating *D. immitis* antigen (6.70%; CI 95%, 3.47–11.71%) in the red foxes population. Comparable results were reported for *A. phagocytophilum* in 16.6% of foxes in central Italy [73] and in 8.2% in Germany [32] and for *D. immitis* in 11% of red foxes from Spain [74]. In contrast, a much lower prevalence of 1 to 3% was found in Sicily [75], Poland [76], and Romania [33]. In Croatia, studies on vector-borne diseases in domestic dogs [55,56,57] have been documented, but studies on wild animals are very rare [77]. Therefore, this study is the first molecular detection of *A. phagocytophilum* in the blood of red foxes from Croatia. The detection of *A. phagocytophilum* antibodies as well as circulating *D. immitis* antigen in moderate proportions in the study population indicates that the foxes were exposed to the pathogens. The foxes were 1.63 times more likely to be exposed to *Anaplasma* than *D. immitis*, but this difference was not statistically significant (*p* = 0.2526; χ^2^ = 1.309). The molecular identification of *A. phagocytophilum* could also confirm the role of red foxes in maintaining the silvatic cycle of these pathogens in Croatia and, from an epidemiological point of view, the role of foxes as potential reservoirs for dogs. Although Lyme borreliosis in humans [52] is known to occur in northern Croatia, the role of foxes in the maintenance and spread of Lyme borreliosis in Croatia has not been investigated. Although one sample was seropositive for B. burgdorferi, DNA was not detected in any of the samples.

The prevalence rates varied according to geographical region, age, and sex. A comparison of geographical regions shows that *Anaplasma* antibodies were found in all districts except near the capital Zagreb (City of Zagreb and Krapina-Zagorje County), while *D. immitis* was found at a higher percentage in this area. It is also striking that *D. immitis* was either found with a significantly low prevalence in the northern area (Zagreb County) or not found at all in the counties bordering Hungary (Varaždin, Međimurje, and Bjelovar-Bilogora Counties) (Table 1, Figure 1). Despite the lack of statistically significant differences, it is noteworthy that the highest number of positive foxes was found in Karlovac County, which is located in the southern study area and has the largest water areas according to the Water Area Management Plan 2022–2027 [78]. The importance of environmental factors, such as water bodies as risk factors for vector-borne diseases, was also emphasised by Gortázar et al. [74], who reported a higher prevalence of *D. immitis* in red foxes in areas along large rivers in north-eastern Spain. These regions could serve as reservoirs for certain helminth species for domestic dogs or wolves. Regarding sex, a slightly higher prevalence and odds ratio for *Anaplasma* antibodies and *D. immitis* antigen was found in female foxes (Table 2 and Table 3). However, this difference was not statistically significant (*p* = 0.0673), which is consistent with the results obtained in foxes in Serbia [79] and in dogs in Italy [80]. When comparing the prevalence as a function of age structure, *D. immitis* was found seven times more frequently in young foxes (*p* < 0.05), which is consistent with the results from Spain [74] and Italy [81]. However, although there was a significant difference in prevalence, no statistically significant association was found between age and exposure to *D. immitis* (OR = 7.75; CI 95%, 0.94–63.79; *p* = 0.057) (Table 2). Comparing the results of this study on foxes with data from studies on the dog population in Croatia [55,56,57,58], the prevalence was higher in foxes. Although no direct link between foxes and the transmission of anaplasmosis and dirofilariasis to dogs was found in this study, the possibility that foxes play a role in maintaining the infection cycle in certain habitats (e.g., hunting areas) cannot be ruled out—especially in areas where infected foxes have been detected. These habitats are often close to or even within human settlements, where dogs are at higher risk of encountering infected vectors. Despite some limitations that may arise from the method of sampling (part of the oral rabies vaccination monitoring programme), such as seasonality (sampling may not have been evenly distributed throughout the year, allowing some seasonal effects to go unnoticed) and the passive nature of sampling (sampling was not systematically planned), the data can still provide valuable insights into the fox population in the region due to the wide territorial coverage and relative randomness of the sampling.

The positive results for *Anaplasma* antibodies indicate exposure in foxes, which was also confirmed by the detection of *A. phagocytophilum* 16S rRNA and GroEL genomes in blood samples from foxes. Although the positive PCR results were found only in blood samples from four foxes (from four counties—Zagreb, Karlovac, Međimurje, and Požega-Slavonia), these results confirm the presence of *A. phagocytophilum* infection in red foxes in Croatia. Serologically positive foxes with negative PCR results can be explained by the fact that the SNAP ^®^ 4Dx ^®^ Plus test detects antibodies that develop after bacteraemia and can be detected in the late phase of the infection, when the pathogen is no longer detectable in the blood by PCR, as also emphasised by Cardoso at al [82] and Lara et al. [83]. On the other hand, sometimes it was not possible to detect or isolate the pathogen due to the characteristics of the sample (which came from a dead animal). Our study therefore shows that commercially available rapid tests based on antibody detection in samples from dead animals can be used to screen and monitor the occurrence of *A. phagocytophilum*. In the case of a seronegative but PCR-positive sample, PCR is useful to detect the possible early phase of infection, when antibody levels are low or undetectable.

The foxes that reacted positively to the *D. immitis* antigen with the SNAP ^®^ 4Dx ^®^ Plus test were PCR-negative, which can be explained by the high sensitivity of the SNAP ^®^ 4Dx ^®^ Plus test for the detection of *D. immitis* antigen, as the test detects the presence of adult forms of *D. immitis* in the blood, which is consistent with the studies by Liu et al. [84] and Henry et al. [85], who found that the sensitivity of the SNAP ^®^ 4Dx ^®^ Plus test for *D. immitis* antigen was 4.1% and 99.18%, respectively. On the other hand, insufficient and autolysed samples or insufficient sample quantities to detect the required DNA fragments in the blood, followed by a low number of microfilariae, can lead to a negative molecular result. Similar results were obtained in foxes [86], cats [87], and dogs [88]. A higher prevalence of *D. immitis* was found in animals with a SNAP antigen test, but no DNA molecules and microfilariae were found in the blood. The samples were also taken from dead animals, which may also have interfered with the detection of microfilariae. Potkonjak et al. [38] and Albonico et al. [89] also reported false-negative PCR test results using the standard PCR protocol, while the same authors confirmed the presence of *D. immitis* DNA in the same samples using more specific and sensitive real-time PCR tests. While the autolytic process may also affect other pathogens, intracellular organisms such as *Anaplasma* spp. may be less affected due to their protected location within host cells. Importantly, SNAP ^®^ tests detect circulating antigens, which are more stable and can remain detectable despite autolysis. A positive antigen result therefore indicates infection and suggests that foxes may serve as a reservoir, even if PCR tests are negative; however, further studies are needed to clarify their role in transmission.

The lack of PCR tests for *Ehrlichia* spp., *A. platys*, and *B. burgdorferi* strains not belonging to OspA could be a limiting factor in our study. Although the SNAP ^®^ 4Dx ^®^ Plus test provides valuable screening data, serological results without molecular confirmation should be interpreted with caution. Although the SNAP ^®^ 4Dx ^®^ Plus test is a convenient and standardised method for detecting circulating antigens and antibodies against multiple pathogens, its sensitivity and specificity may vary depending on the stage of infection, pathogen load, and host immune response. While positive results indicate exposure or infection, they should be interpreted with caution, especially in the absence of confirmatory molecular testing. This limitation is particularly important in epidemiological studies and was taken into account in the interpretation of our results.

Although discrepancies between serological and molecular results may indicate false-positive results, these results reflect the inherent strengths and limitations of each method. Serological tests such as SNAP ^®^ 4Dx ^®^ Plus are highly sensitive for detecting antibodies and antigens, especially in later stages of infection, while PCR is more effective in early stages of infection when the pathogen DNA is present in the blood. Given these dynamics, we recommend a combined diagnostic approach—using serology for broad surveillance and PCR for confirmation—to increase accuracy and better understand the epidemiology of vector-borne pathogens in wildlife.

Considering the known epidemiological data for *Erlichia* and *Borrelia*, in Croatia, the negative result for *Erlichia* was to be expected, as *Ehrlichia* is currently not considered a major problem in Croatia. In previous studies, antibodies were detected only in a very small proportion of dogs (up to 0.6%) [55,56], and molecular detection of *E. canis* was only recently achieved in the tick *Rhipicephalus bursa* [90], and no data are available for humans either. The fact that we could not detect *Borrelia* is somewhat surprising considering that a higher percentage of Lyme borreliosis has been detected in humans [48], but on the other hand this can also be explained by the fact that a low seroprevalence has been found in animals [55,56,57].

The results of this study provide important insights into the epidemiology of vector-borne pathogens in red foxes in northern Croatia. The detection of *A. phagocytophilum* and *D. immitis* in moderate proportions in several districts emphasises the geographical distribution and potential endemicity of these pathogens in wild carnivore populations. Detection of *A. phagocytophilum* DNA, including 16S rRNA and groEL gene fragments, confirms active infection and contributes to understanding the genetic diversity of circulating strains. Although further phylogenetic analyses are required, these molecular findings indicate the presence of potentially zoonotic variants and support the hypothesis of cross-species transmission. The spatial distribution of positive cases, particularly in areas with many bodies of water and in the immediate vicinity of human settlements, emphasises the ecological factors that influence the spread of the pathogen. Due to their adaptability to periurban environments and their exposure to vector habitats, red foxes may serve as sentinel species or even reservoirs for certain vector-borne pathogens and pose a potential risk to domestic animals and humans. These findings emphasise the need for integrated surveillance strategies that include wildlife surveillance as part of a One Health approach to vector-borne disease control.

## 5. Conclusions

To summarise, of the vector-borne zoonoses investigated in the red fox population in Croatia, the results indicate the presence of infections of the species *D. immitis* and *A. phagocytophilum*, while infections of Lyme disease and ehrlichiosis could not be confirmed. This study is the first molecular detection of *A. phagocytophilum* in the blood of red foxes from Croatia. It also shows that commercially available rapid tests based on antibody detection in samples from dead animals can be used to screen for *A. phagocytophilum* and *D. immitis*. Our results also indicate a higher prevalence of *A. phagocytophilum*, and significant differences were found between geographical areas (districts) and between age groups. The results suggest that *Anaplasma* and *Dirofilaria* infections persist in the red fox population in the areas studied, emphasising their potential role in maintaining the sylvatic cycle of these pathogens, which is of considerable public and veterinary health importance. Despite the molecular detection of *A. phagocytophilum* in the blood of red foxes in Croatia, and given the ecological adaptability of red foxes and their frequent presence in the vicinity of human settlements, their role as sentinel or reservoir hosts warrants further investigation. The combined use of serological and molecular diagnostics proved valuable for detecting the different stages of infection, although the limited sensitivity of PCR suggests that more advanced techniques such as real-time PCR are required. Future studies should aim to expand geographic and seasonal coverage, include vector samples, and perform genetic characterisation of detected pathogens to better understand transmission dynamics and inform One Health surveillance strategies.

## Figures and Tables

**Table 1:** Detailed results of the screening test for *D. immitis* and *Anaplasma* in red foxes by geographical area.

County	Foxes Examined	Positive for *D. immitis* Antigen	CI 95%	Positive for *Anaplasma* Antibody	CI 95%	*p*-Value
	N	%		%		
Zagreb	53	1.89 *	0.05–10.51	11.32	4.15–24.64	0.0506
Krapina-Zagorje	17	11.76	1.42–42.50	0	0	0.1450
Sisak-Moslavina	21	14.29	2.95–41.75	4.76	0.12–26.53	0.2928
Karlovac	26	15.39	4.19–39.39	23.08	6.89–39.28	0.4818
Varaždin	13	0	0	7.69	0.16–42.86	0.3079
Bjelovar-Bilogora	4	0	0	25.00	0.63–100.00	0.2850
Požega-Slavonia	22	4.55	0.12–25.33	4.55	0.16–25.33	1.00
Međimurje	19	0	0	10.53	1.27–38-02	0.1462
City of Zagreb	4	25.00	0.63–100.00	0	0	0.2850
Total	179	6.70	3.47–11.71	10.06	5.96–15.89	0.063

* Statistically significant, *p* < 0.05.

**Table 2:** Association of positive findings for *D. immitis* in foxes with area, sex, and age (OR assessment).

Risk Factor	Variable	Foxes Examined (N)	Prevalence (%)	OR ^1^	CI 95% ^2^	*p*-Value
County	Zagreb Krapina-Zagorje Sisak-Moslavina Karlovac Požega-Slavonia Međimurje	53 17 21 26 22 19	1.89 11.76 14.29 15.39 4.55 10.53	reference value 6.93 8.21 9.45 2.48 6.12	0.59–81.83 0.80–83.83 0.99–89.46 0.15–41.45 0.52–71.76	0.1241 0.0757 0.0501 0.5282 0.1494
Sex	male female	108 67	6.48 7.46	reference value 2.28	0.69–7.50	0.1746
Age	adult juvenile	62 71	1.61 11.27	reference value 7.75	0.94–63.79	0.0570

^1^ Odds ratio; ^2^ confidence interval for OR.

**Table 3:** Association of positive findings for *A. phagocytophilum* in foxes with area, sex, and age (OR assessment).

Risk Factor	Variable	Foxes Examined (N)	Prevalence (%)	OR ^1^	CI 95% ^2^	*p*-Value
County	Zagreb Krapina-Zagorje Sisak-Moslavina Karlovac Požega-Slavonia Međimurje	53 17 21 26 22 19	4.55 11.32 4.76 25.08 7.69 10.53	reference value 2.68 1.05 6.30 1.75 2.47	0.31–23.68 0.06–17.94 0.70–57.08 0.10–30.59 0.21–29.63	0.3750 0.9731 0.1016 0.7015 0.4755
Sex	male female	108 67	6.48 7.46	reference value 2.53	0.91–7.01	0.0740
Age	adult juvenile	62 71	9.86 12.90	reference value 0.75	0.26–2.21	0.6037

^1^ Odds ratio; ^2^ confidence interval for OR.

## Data Availability

All relevant data are provided in the manuscript. Further inquiries can be directed to the corresponding author.
